# *Aikeqing* decreases viral loads in SHIV89.6-infected Chinese rhesus macaques

**DOI:** 10.1186/s13020-016-0105-x

**Published:** 2016-07-01

**Authors:** Gao-Hong Zhang, Jian-Bao Han, Lin Zhu, Rong-Hua Luo, Xi-He Zhang, Xin Chen, Ying-Jie Hu, Lin-Chun Fu, Yong-Tang Zheng

**Affiliations:** Key Laboratory of Animal Models and Human Disease Mechanisms of the Chinese Academy of Sciences and Yunnan Province, Kunming Institute of Zoology, Chinese Academy of Sciences, Kunming, 650223 Yunnan China; Kunming College of Life Science, University of Chinese Academy of Sciences, Kunming, 650204 Yunnan China; Institute of Tropical Medicine, Guangzhou University of Chinese Medicine, Guangzhou, 510006 China; Kunming Primate Research Center of the Chinese Academy of Science, Kunming Institute of Zoology, Chinese Academy of Sciences, Kunming, 650223 Yunnan China

## Abstract

**Background:**

*Aikeqing* (AKQ) has been shown in clinical studies to improve quality of life of HIV/AIDS patients, but anti-HIV activity has not been determined. The SHIV-infected macaque is an important animal model for testing antiviral drugs. This study aimed to determine the anti-HIV activity of AKQ in chronically SHIV89.6-infected Chinese rhesus macaques.

**Methods:**

Nine Chinese rhesus macaques were inoculated intravenously with SHIV89.6 virus. At 11 weeks post-infection, the animals were arbitrarily divided into three groups: high-dose (AKQ 1.65 g/kg; *n* = 3), low-dose (AKQ 0.55 g/kg; *n* = 3), and control (water 1 mL/kg; *n* = 3). Treatment was administered by the intragastric gavage route once-daily for 8 weeks. Blood (5 mL) was collected biweekly. Viral loads were analyzed by real-time quantitative RT-PCR assays, and T cell counts were monitored by FACS analyses throughout the treatment.

**Results:**

AKQ induced a persistent decline (*P* = 0.02) in plasma viral loads during treatment in the high-dose group compared with their baseline levels, and cessation of the therapy caused viral load rebound to the pretreatment levels. No significant difference (*P* = 0.06) was found in the plasma viral loads during treatment in the low-dose group. The CD4^+^ T cell counts and CD4/CD8 ratios remained at stable high levels during the treatment period.

**Conclusion:**

AKQ reduced plasma viral loads in the SHIV89.6-infected Chinese rhesus macaque model.

**Electronic supplementary material:**

The online version of this article (doi:10.1186/s13020-016-0105-x) contains supplementary material, which is available to authorized users.

## Background

Highly active antiretroviral therapy (HAART) has significantly improved the morbidity and mortality of HIV-infected patients. A total of 37 licensed drugs are available for optimal antiretroviral combinations [1]. However, not all patients can profit from long-term therapy, and patients may still have an increased risk of death from non-AIDS-associated malignancies. Most patients receiving HAART have some persistent immune dysfunction [2]. Non-AIDS-associated malignancies are becoming a large burden on the healthcare system owing to the improved survival of HIV-infected patients receiving HAART.

Increasing evidence has demonstrated that drugs targeting multiple targets of HIV-1 infection usually have amplified therapeutic efficacy [3]. *Aikeqing* (AKQ), composed of Radix *Aconiti Lateralis Preparata* (*Fu Zi*), *Herba Epimedii* (*Yin Yang Huo*), Rhizoma *Zingiberis* (*Gan Jiang*), Radix *Glycyrrhizae* (*Gan Cao*), Radix *Ginseng* (*Ren Shen*), Radix *Salviae Miltiorrhizae* (*Dan Shen*), Rhizoma *Polygoni Cuspidati* (*Hu Zhang*), *Poria* (*Fu Ling*), Cortex *Phellodendri* (*Huang Bai*), and Radix *Scutellariae* (*Huang Qin*), was designed based on the symptoms and characteristics of HIV/AIDS patients by investigators at the Institute of Tropical Medicine, Guangzhou University of Chinese Medicine [4]. Pilot clinical studies [4, 5] reported beneficial effects of AKQ in the treatment of HIV patients in China, by improving their quality of life and reducing side effects related to medications. However, the HIV loads and CD4^+^ T cell counts, which are routinely measured to monitor HIV disease progression, were not determined in the treated patients because of the associated costs and technical constraints.

Rhesus macaques closely resemble humans in anatomy, physiology, drug metabolism, and other characteristics. Moreover, their susceptibility to SIV/SHIV infection makes them a superior animal model for testing of anti-HIV drugs. Chinese-origin rhesus macaques (Ch-RMs) are the most appropriate species for studying SIV/SHIV infection and AIDS pathogenesis [6, 7]. To our knowledge, there are no reports on the use of SHIV-infected Ch-RMs to test the efficacy of a Chinese medicine.

This study aimed to determine the anti-HIV activity of AKQ in chronically SHIV89.6-infected Ch-RMs.

## Methods

### Herbal materials and AKQ extraction

AKQ was provided by the Tropical Medicine Institute, Guangzhou University of Chinese Medicine, and composed of Radix *Aconiti Lateralis Preparata*, *Herba Epimedii*, Rhizoma *Zingiberis*, Radix *Glycyrrhizae*, Radix *Ginseng*, Radix *Salviae Miltiorrhizae*, Rhizoma *Polygoni Cuspidati*, *Poria*, Cortex *Phellodendri*, and Radix *Scutellariae* at ratios of 10:5:5:5:3:3:3:3:1.5:1.5 (Table 1). All air-dried herbs in AKQ are regionally well-known herbal medicines that are commercially used in China, and were purchased from Guangzhou Medicine Company (Guangzhou, China). The authenticity was identified by herbalists within that company, and re-confirmed by Prof. Ying-Jie Hu through morphological and anatomical identifications. The voucher specimens (AKQ HM-1–AKQ HM-10) were deposited at the Tropical Medicine Institute, Guangzhou University of Chinese Medicine.

**Table 1:** Composition of *Aikeqing*

Chinese name	English name	Latin name	Part used	Composition ratio
Fuzi	Radix AconitiLateralisPreparata	Aconitum carmichaeli Debx.	Black slice of root	10
Yinyanghuo	Herba Epimedii	Epimedium brevicornum Maxim.	Overground portion	5
Ganjiang	Rhizoma Zingiberis	Zingiber officinaleRosc.	Rhizoma	5
Gancao	Radix Glycyrrhizae	Glycyrrhiza uralensis Fisch.	Root and Rhizoma	5
Renshen	Radix Ginseng	Panax ginseng C. A. Mey.	Root	3
Danshen	Radix SalviaeMiltiorrhizae	Salvia miltiorrhizaBge.	Root	3
Huzhang	Rhizoma Polygoni Cuspidati	Polygonum cuspidatum Sieb. etZucc.	Root	3
Fuling	Poria	Poriacocos (Schw.) Wolf.	Sclerotium	3
Huangbai	Cortex Phellodendri	Phellodendron chinensis Schneid.	Bark	1.5
Huangqin	Radix Scutellariae	Scutellaria baicalensis Georgi	Root	1.5

The raw herbal materials were mixed and extracted in 20 volumes of boiling water for 2 h, with the extraction repeated three times. The aqueous extracts were filtered and the suspension was collected. The filtrate was added to 2 volumes of 95 % (v/v) ethanol. The two suspensions were mixed thoroughly and left to stand for 10 h. The second mixture was filtered and the suspension was collected. The extraction solution was concentrated by stove-drying the residue, smashed to a fine powder, and stored in a desiccator until use.

### Animals and study design

The nine juvenile male Ch-RMs (age: 3–6 years; body weight: 5.5–10.2 kg) used in this study were colony-bred rhesus macaques (*Macaca mulatta*) of Chinese origin that were maintained in accordance with the regulations of the American Association for Assessment and Accreditation of Laboratory Animal Care at the Kunming Primate Research Center, Kunming Institute of Zoology, Chinese Academy of Sciences. All experimental procedures were performed at the ABSL-3 Laboratory according to the guidelines approved by the Ethics Committee of Kunming Institute of Zoology (Approval Number: SWYX-2011020; Additional file 1). Room temperature of the ABSL-3 Laboratory was maintained at 20–28 °C, with a relative humidity of 35–60 % and a 12-h/12-h light/dark cycle. The Ch-RMs were housed in stainless-steel cages (800 × 1000 × 1000 mm), and fed a monkey diet as well as fresh fruits, vegetables, and nuts. All animals had free access to feed and water ad libitum. The general conditions of the Ch-RMs, such as health status, behavior, eating habits, and stools, were observed daily by a veterinarian. The animal studies were performed following the ARRIVE guideline (Additional file 2).

All animals were demonstrated to be seronegative for SIVmac, simian T cell leukemia virus type 1, simian retrovirus type 1 (type D retrovirus), and herpesvirus B. To allow for achievement of steady-state parameters, treatment was initiated at week 11 in the early phase of chronic infection and lasted for 8 weeks thereafter. Blood samples were obtained biweekly during the infection. One animal (#02067) in the control group died at the initiation of therapy (day 5), suggesting that its immune response might not have been sufficient for protection against SHIV infection. All data from week 12 onward refer to a total of two animals in the control group.

### SHIV infection and treatment

The Ch-RMs were inoculated intravenously with 1000 TCID_50_ (50 % tissue culture infectious doses) of cell-free SHIV89.6 grown in CEM × 174 cells. The animals were arbitrarily divided into three groups according to the cage placement within rooms in the animal house: control group (water 1 mL/kg): #02067, #06003, #06025; AKQ high-dose group (AKQ 1.65 g/kg): #03057, #04331, #06403; and AKQ low-dose group (AKQ 0.55 g/kg): #01089, #05077, #06311. AKQ was administered to the Ch-RMs once-daily by the intragastric route through a nasogastric tube (Sofine, Zhejiang, China).

### Plasma collection

For blood collections, the animals were immobilized with ketamine-HCl (10 mg/kg body weight) injected intramuscularly at the lateral thigh muscles. Peripheral blood (5 mL) was collected into vacutainer tubes (Zhiyuan, Wuhan, China) containing EDTA-K_2_ as an anticoagulant. Blood was collected from the lateral aspect of the hind limb. Plasma was separated by centrifugation of the blood in an Allegra X-12 centrifuge (Beckman Coulter, Fullerton, CA, USA) at 900×*g* for 10 min at 4 °C, and stored at −80 °C until further processing and analysis.

### Hematological parameter assays

Aliquots of blood (100 μL) were transferred to tubes for hematological analyses. Hematological parameters were measured using an automated hematological analyzer machine (BC-2800Vet; Mindray, Shenzhen, China). The abbreviations and units of the hematologic parameters are described in Table 2.

**Table 2:** Hematological parameters of SHIV89.6-infected Ch-RMs during *Aikeqing* treatment

Markers	Group	Weeks post administration of AKQ		
		0	2	4
WBC	H	7.2 ± 2.0	8.1 ± 0.8	7.2 ± 1.1
	L	7.0 ± 1.6	8.2 ± 0.4	8.4 ± 0.6
	C	7.6 ± 1.9	8.0 ± 2.0	7.3 ± 2.3
Mon (%)	H	7.3 ± 0.7	8.1 ± 2.2	7.7 ± 2.1
	L	7.9 ± 0.9	8.9 ± 1.6	7.6 ± 0.9
	C	7.6 ± 1.1	8.1 ± 1.3	8.2 ± 0.3
Mon (109/L)	H	0.4 ± 0.1	0.5 ± 0.1	0.4 ± 0.2
	L	0.4 ± 0.2	0.4 ± 0.4	0.4 ± 0.2
	C	0.6 ± 0.3	0.4 ± 0.3	0.7 ± 0.2
RDW (%)	H	15.6 ± 0.3	16.4 ± 0.4	15.5 ± 0.1
	L	15.8 ± 0.3	16.1 ± 0.3	15.5 ± 0.8
	C	14.7 ± 0.6	16.2 ± 0.2	15.5 ± 1.0
RBC (10^12^/L)	H	7.5 ± 0.4	6.6 ± 0.4	6.8 ± 0.1
	L	7.1 ± 0.4	5.6 ± 0.4	6.0 ± 0.6
	C	7.1 ± 0.3	6.0 ± 0.9	5.8 ± 0.8
HCT (%)	H	58.7 ± 2.0	51.2 ± 3.4	53.9 ± 1.0
	L	53.2 ± 2.9	42.8 ± 3.2	46.7 ± 4.9
	C	52.9 ± 2.4	46.0 ± 7.2	45.6 ± 7.0
Lym (10^9^/L)	H	1.7 ± 0.3	2.0 ± 0.2	2.5 ± 0.5
	L	1.5 ± 0.2	1.6 ± 0.6	2.0 ± 0.3
	C	1.8 ± 0.5	1.8 ± 0.7	2.0 ± 0.3
Lym (%)	H	22.4 ± 6.0	27.3 ± 10.5	36.9 ± 12.5
	L	19.0 ± 9.7	17.4 ± 5.9	34.0 ± 7.8
	C	22.8 ± 12.4	23.2 ± 22.0	32.7 ± 13.9
MCV (fL)	H	74.6 ± 0.2	77.5 ± 0.5	79.0 ± 0.3
	L	75.0 ± 0.5	76.9 ± 0.4	78.6 ± 0.2
	C	74.3 ± 0.5	77.3 ± 0.8	78.5 ± 1.5
MCH (pg)	H	24.4 ± 0.4	25.6 ± 0.6	26.8 ± 0.5
	L	23.9 ± 1.2	25.0 ± 0.3	26.2 ± 0.2
	C	22.9 ± 1.9	24.1 ± 1.9	24.2 ± 2.4
MCHC (g/L)	H	328.3 ± 4.2	331.3 ± 7.8	339.3 ± 6.7
	L	319.3 ± 13.9	326.0 ± 4.4	333.3 ± 3.2
	C	309.0 ± 23.4	311.7 ± 22.0	308.3 ± 26.5
MPV (fL)	H	7.2 ± 0.1	7.8 ± 0.6	8.0 ± 0.5
	L	7.3 ± 0.1	7.9 ± 0.4	8.1 ± 0.3
	C	6.9 ± 0.5	8.1 ± 0.5	7.6 ± 0.5
HGB (g/L)	H	189.7 ± 5.0	176.3 ± 4.5	183.0 ± 1.0
	L	170.0 ± 9.6	149.7 ± 8.1	156.0 ± 15.6
	C	160.7 ± 14.2	158.0 ± 24.5	152.0 ± 24.8
PDW	H	16.5 ± 0.1	16.6 ± 0.3	16.6 ± 0.2
	L	16.8 ± 0.5	16.3 ± 0.2	16.3 ± 0.2
	C	16.6 ± 0.1	16.3 ± 0.2	16.2 ± 0.2
PLT (10^9^/L)	H	410.0 ± 76.3	367.0 ± 60.9	287.7 ± 27.1
	L	331.7 ± 27.7	440.3 ± 67.3	372.7 ± 61.2
	C	418.0 ± 25.0	459.7 ± 24.0	377.7 ± 51.9
PCT (%)	H	0.3 ± 0.1	0.3 ± 0.0	0.2 ± 0.0
	L	0.2 ± 0.1	0.4 ± 0.1	0.3 ± 0.1
	C	0.2 ± 0.1	0.4 ± 0.0	0.3 ± 0.1
Gran (10^9^/L)	H	3.8 ± 0.7	5.6 ± 4.6	2.9 ± 1.4
	L	2.9 ± 0.2	4.4 ± 2.0	2.2 ± 1.3
	C	2.4 ± 0.8	4.6 ± 1.5	2.7 ± 0.7
Gran (%)	H	71.3 ± 7.1	63.9 ± 6.6	58.1 ± 12.6
	L	72.8 ± 8.2	71.0 ± 7.7	60.0 ± 7.5
	C	70.3 ± 14.0	69.6 ± 12.5	64.5 ± 8.4

*H* high-dose group, *L* low-dose group, *C* control group, *WBC* white blood cells, *Mon* monocytes, *RDW* red blood cell volume distribution width, *RBC* red blood cells, *HCT* haematocrit, *Lym* lymphocytes, *MCV* mean corpuscular volume, *MCH* mean corpuscular haemoglobin, *MCHC* mean corpusular hemoglobin concerntration, *MPV* mean platelet volume, *HGB* haemoglobin, *PDW* platelet distribution width, *PLT* platelets, *PCT* plateletcrit, *Gran* granulocytes

### Lymphocyte phenotyping by flow cytometry

Peripheral blood lymphocyte subset analyses were performed in a FACSCalibur flow cytometer (BD, Franklin Lakes, NJ, USA) using a panel of mouse anti-human monoclonal antibodies known to cross-react with macaque receptors. Staining was performed on whole blood preparations. The lymphocyte subsets were stained for CD3 (PE; clone SP34; BD), CD4 (PerCP; clone L200; BD), and CD8 (FITC; clone RPA-T8; BD). The T cell subset counts in the peripheral blood were determined by the TruCount™ method (BD, San Jose, CA, USA) using the FACSCalibur flow cytometer. The CD4^+^ and CD8^+^ T cell counts were reported as cell number/microliter.

### Viral load measurement

Plasma samples were analyzed for viral RNA (vRNA) by real-time quantitative RT-PCR assays as described previously [8]. Briefly, vRNA was extracted with a High Pure Viral RNA Kit (Roche, Mannheim, Germany) in accordance with the manufacturer’s instructions. A two-step RT-qPCR assay using a PrimeScript™ RT Reagent Kit and Premix ExTaq™ (Takara, Dalian, China) was performed in a 7500 Fast Real-Time PCR System (Applied Biosystems, Foster City, CA, USA). Each sample was tested in triplicate. Viral loads were reported as vRNA copy numbers per milliliter of plasma. The detection limit of the assay was 100 vRNA copies/mL.

### Statistical analysis

Statistical analyses were performed using SPSS software 21.0 (SPSS Inc., Chicago, IL, USA) and GraphPad Prism version 6.0 (GraphPad Inc., San Diego, CA, USA). An independent-samples *t*-test was used for comparisons of differences in CD4^+^ T cell counts and plasma viral loads between two independent groups. A paired-samples *t*-test was used to compare CD4^+^ T cell counts and plasma viral loads between different time points within the same group. Spearman’s correlation analysis was used to assess the relationships between CD4/CD8 ratio and CD4^+^ T cell count, CD8^+^ T cell count, or plasma vRNA level. Values of *P* < 0.05 were considered statistically significant.

## Results

### Side effects of AKQ during treatment

All animals had complete blood counts monitored in the early stage of treatment to identify any side effects of AKQ (Table 2). The appetite, stool, and conditions of the animals were observed daily by a veterinarian during treatment. Most hematological parameters remained unchanged, and no obvious side effects were observed in the treated groups after 8 weeks of therapy. The high dose of AKQ (1.65 g/kg body weight) was tolerated in all animals without evidence of drug toxicity, the weights of the Ch-RMs remained relatively constant during AKQ treatment, and no animals were euthanized.

### AKQ treatment reduced plasma viral loads

**Fig. 1:**
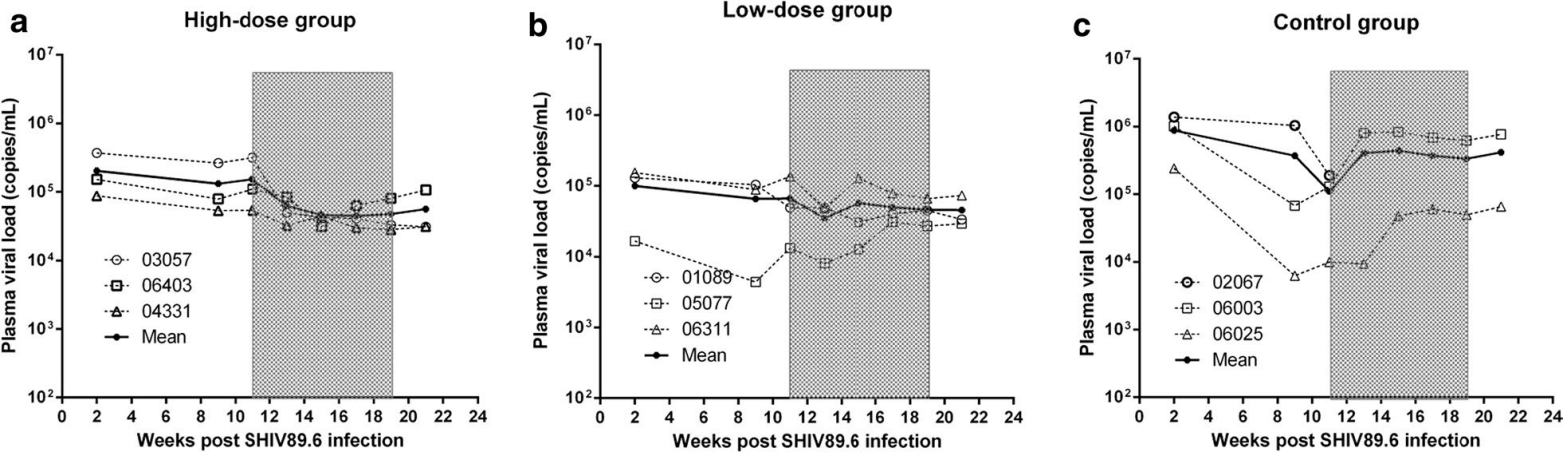
AKQ reduces plasma viral loads in chronically SHIV89.6-infected Ch-RMs. Nine SHIV89.6-infected Ch-RMs were randomly divided into three groups (*n* = 3 per group), and treated with 1.65 g/kg/day AKQ (**a)**, 0.55 g/kg/day AKQ (**b)**, or placebo (**c)** for 8 weeks. The *gray-shaded* areas indicate the treatment period. The *dotted lines* indicate the results obtained from individual animals, and the *solid lines* represent the mean values for the groups. Plasma viral loads were measured by real-time PCR assays with a sensitivity of 100 vRNA copies/mL

Following intravenous infection (week 0), the plasma viral loads were measured biweekly throughout the study. As observed in our previous study [8], all animals became infected with variable peak and set point viral loads (Fig. 1). The majority of the animals maintained viral loads of 10^4^–10^6^ copies/mL for the duration of the experiment. Higher levels of SHIV viremia might have resulted in significant pathogenesis and rapid disease progression in one animal in the control group (#02067), and this animal died at week 12 post-infection.

Comparisons of animals from different groups revealed that treatment with high-dose AKQ induced a persistent decline (*P* = 0.02) in plasma vRNA loads during treatment (Fig. 1). The viral load copy numbers of the three Ch-RMs in the high-dose group decreased by 42–88 % (Fig. 1a), while no significant decrease (*P* = 0.06) in the plasma viral loads was detected in the low-dose group. Ch-RM #01089 (Fig. 1b, c) in the low-dose group also exhibited a sustained decline in viral load from the baseline value of 136,749–47,923 copies/mL at week 2 post-treatment. The animals in the low-dose group and control group maintained persistent viremia compared with those in the high-dose group. The viral loads rapidly rebounded in the treated Ch-RMs following the cessation of treatment. In contrast to the variability of viral loads in the control group, the patterns of viral loads were similar in the high-dose group.

### AKQ therapy transiently increased T cell counts

**Fig. 2:**
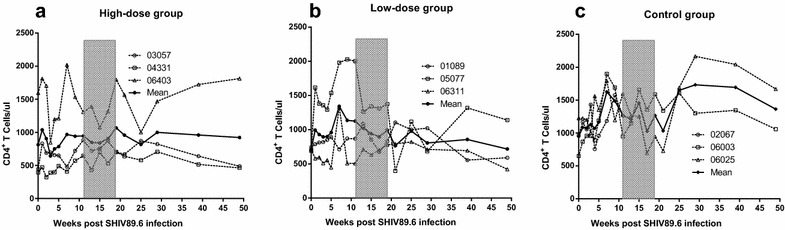
Changes in CD4^+^ T cell counts in peripheral blood. The absolute CD4^+^ T cell counts remained stable or increased after treatment in the high-dose group (**a**), compared with those in the low-dose group (**b**) or control group (**c**). No significant changes were observed for the CD4^+^ T cell counts in the treated and untreated groups before and after treatment

To estimate the effect of AKQ therapy on T lymphocytes, we measured the CD4^+^ and CD8^+^ absolute cell numbers. The CD4^+^ T cell counts showed slight increases or remained stable after AKQ administration in the high-dose group compared with the baseline levels (*P* = 0.50; Fig. 2a) or the low-dose group (*P* = 0.53; Fig. 2b, c). The effect of AKQ treatment on the mean CD4^+^ T cell count was small and non-significant (*P* = 0.39) in the low-dose group. The increases in the peripheral T cell counts following AKQ treatment were transient and decreased rapidly after the end of treatment in the high-dose group.

**Fig. 3:**
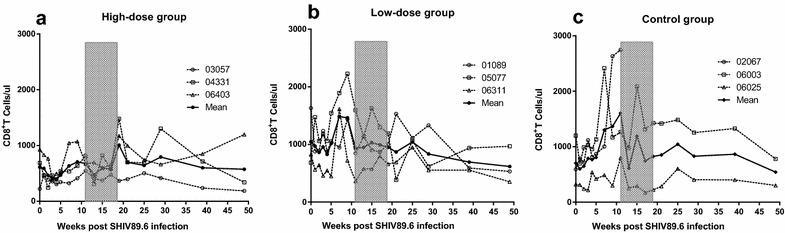
Changes in CD8^+^ T cell counts in peripheral blood. The CD8^+^ T cell counts were assessed in the high-dose group (**a**), low-dose group (**b**), and control group (**c**). No significant changes were observed for the CD8^+^ T cell counts in the treated and untreated groups before and after treatment

The CD8^+^ T cell counts were slightly increased after treatment in the high-dose group (Fig. 3a). No significant change (*P* = 0.57) was observed in the high-dose group compared with the low-dose group (Fig. 3b, c).

### Effect of AKQ treatment on the CD4/CD8 ratio

**Fig. 4:**
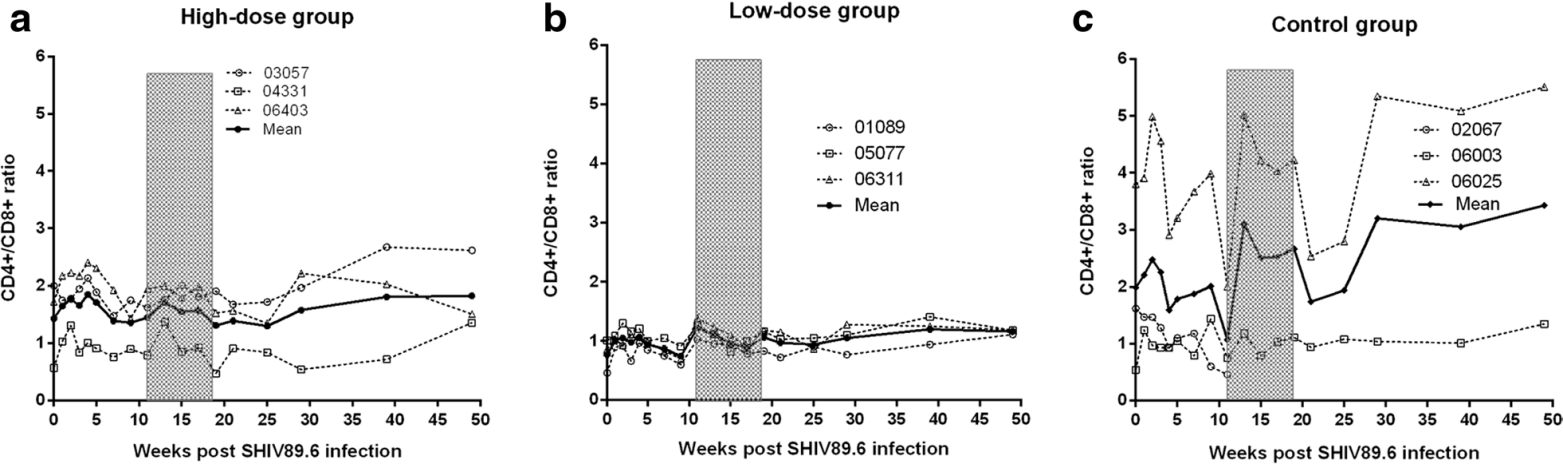
Effect of treatment on peripheral blood CD4/CD8 T cell ratios. The CD4/CD8 T cell ratio remained at a consistently high level in the high-dose group (**a**), low-dose group (**b**), and control group (**c**) during the treatment. No significant changes were observed in the treated and untreated groups before and after treatment

**Fig. 5:**
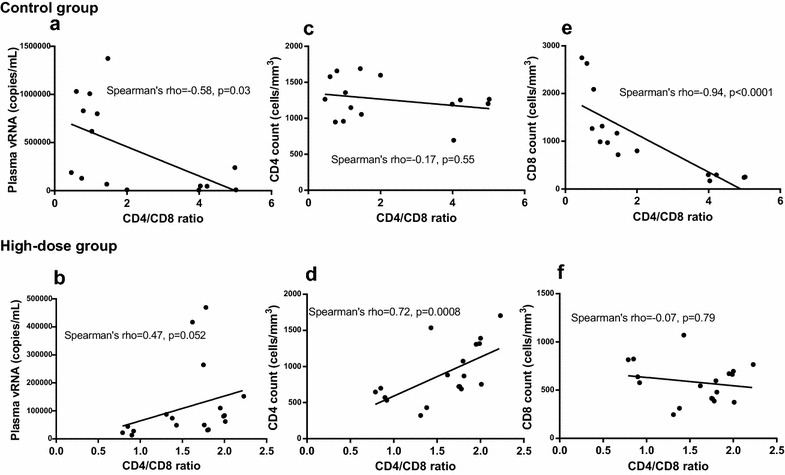
Correlations of the CD4/CD8 ratio with the CD4^+^ T cell count, CD8^+^ T cell count, or plasma viral load in the treated or untreated Ch-RMs. Spearman’s correlations were analyzed in the control group (*top panel*) and high-dose group (*bottom panel*). The CD4/CD8 ratio was negatively correlated with the plasma viral load in the control group (**a**), but no correlation was found in the high-dose group (**b**). The CD4/CD8 ratio was positively correlated with the CD4^+^ T cell count in the high-dose group (**d**), but no correlation was found in the control group (**c**). The CD4/CD8 ratio was negatively correlated with the CD8^+^ T cell count in the control group (**e**), but no correlation was observed in the high-dose group (**f**)

All animals had a persistently high CD4/CD8 ratio (>1) after SHIV89.6 infection, and no significant difference (*P* = 0.24) was found between the high-dose group and the low-dose group (Fig. 4). Interestingly, further analysis indicated that the CD4/CD8 ratio in the control group showed negative correlations with the viral load and CD8^+^ T cell count (*rho* = −0.58, *P* = 0.03 and *rho* = −0.94, *P* < 0.0001, respectively), but not with the CD4^+^ T cell count (*rho* = −0.17, *P* = 0.55). The findings of the present study were consistent with those in a previous study by Serrano-Villar et al. [9]. However, the results for the high-dose group were quite different. In the high-dose group, a positive correlation was found between the CD4/CD8 ratio and the CD4^+^ T cell count (*rho* = 0.72, *P* < 0.01), and no significant correlations were found between the CD4/CD8 ratio and the CD8^+^ T cell count or viral load (Fig. 5). These results suggest that treatment with AKQ resulted in an increase in CD4^+^ T lymphocytes. The high CD4^+^ T cell count and consequently the high CD4/CD8 ratio might suggest successful recovery of CD4^+^ T cells.

## Discussion

The present study analyzed the effects of AKQ administration on viral loads, CD4^+^ T cell counts, and CD8^+^ T cell counts in SHIV89.6-infected Ch-RMs. To our knowledge, this is the first attempt to study the effect of a Chinese medicine in SHIV89.6-infected Ch-RMs. Ch-RMs develop AIDS-like symptoms more slowly than Indian macaques when exposed to SIV or SHIV [10]. Mothé et al. [11] reported Ch-RM MHC immunogenetics are more similar to HLA than those of Indian macaques, and therefore that Ch-RMs might be a better model for HIV. The SIV-infected Ch-RM has been successfully used as a model for studying responses to combination antiretroviral therapy (ART) and evaluating candidate eradication strategies to cure HIV infection [12]. Our study was performed to develop a chronically SHIV89.6-infected Ch-RM model for detailed investigation of Chinese medicine therapeutic effects. High-dose AKQ therapy reduced the viral loads in SHIV89.6-infected Ch-RMs when initiated early after infection, thereby preventing the immunologic damage caused by HIV-1. AKQ treatment showed a beneficial effect on T cell recovery in the SHIV89.6-infected animals in accordance with previous findings in a clinical study [5].

In untreated HIV-infected patients, the CD4/CD8 ratio can be used not only as a predictor of poor prognosis [13], but also as a predictor of immune restoration [14] and AIDS-related morbidities. Serrano-Villar et al. [9] reported that patients with early initiation of ART were more likely to achieve a higher CD4/CD8 ratio than patients with later initiation of ART (2 years after infection). To our knowledge, no studies have specifically addressed the clinical significance of the CD4/CD8 ratio in SHIV-infected Ch-RMs on therapy. In our study, the animals in the high-dose group displayed a CD4/CD8 ratio increase from their baseline level before AKQ initiation. Meanwhile, a significant increase in the CD4/CD8 ratio failed to appear because the increase in CD4^+^ T cells was accompanied by a simultaneous increase in CD8^+^ T cells. In the untreated animals, the CD4/CD8 ratio showed strong correlations with the CD8^+^ T cell count and plasma vRNA level, but not with the CD4^+^ T cell count. Remarkably, the associated relationships changed and only the CD4^+^ T cell count was positively correlated with the CD4/CD8 ratio in the high-dose group after treatment. We assumed that the CD4/CD8 ratio was essentially dependent on the CD4^+^ T cell count in the animals with successful CD4^+^ T cell recovery. Helleberg et al. [15] reported that a major decline in the CD4^+^ T cell count was associated with markedly increased risks of cardiovascular disease, cancer, and death among ART-treated HIV patients. Takuva et al. [16] reported that patients on ART with poor CD4^+^ T cell recovery during early treatment were at greater risk of progression to new AIDS diagnosis or death despite viral suppression.

Baicalein and baicalin, isolated from *Scutellaria baicalensis* Georgi, were shown to be potent inhibitors of integrase and reverse transcriptase from HIV-1 [17]. Liu et al. [18] reported that crude extracts from Cortex *Phellodendri* exhibited antifungal activities in vitro, and suggested their use for treatment of candidiasis in HIV/AIDS patients. Tang et al. [19] reported that a crude extract of *Rhizoma Polygoni Cuspidati* inhibited HIV-1 reverse transcriptase. Abd-Elazem et al. [20] reported that water-soluble extracts of *Salvia miltiorrhiza Bge.* inhibited HIV-1 integrase activities. All of these findings suggest that AKQ may suppress viral replication at multiple steps in the life cycle of HIV-1.

Many people living with HIV/AIDS in China are using complementary medicine [21]. In 1996, a randomized trial tested a Chinese herbal formulation composed of 31 Chinese herbs in 30 HIV patients for treatment of HIV-related symptoms for a duration of 12 weeks. The study found that the symptoms were reduced in patients receiving the herbal formulation, but not in those receiving the placebo [22]. However, no significant differences were found between the two groups in a follow-up study [23]. A recent study showed that indirubin, the active ingredient of the Chinese herbal formula *Dan Gui Long Hui Wan*, suppressed viremia of multidrug-resistant HIV molecular clones in animal models [24]. HAART for HIV/AIDS is currently limited by the inability to eliminate the latent viral reservoir. The goal of HIV therapy is immune control of virus replication, rather than eradication. A combination of anti-inflammatory, antiretroviral, and immunological therapies might lead to total viral suppression and restoration of immune function in AIDS patients [25, 26]. AKQ at an appropriate concentration (1.65 g/kg) decreased the plasma viral loads in SHIV89.6 chronically-infected Ch-RMs. AKQ was effective in preventing the decline of circulating CD4^+^ T cells. The largest increase in CD4^+^ T cells after treatment was observed in the high-dose group, which corresponded well with the significant decrease in the SHIV89.6 viral loads in this group.

One possible limitation of the present study was the small number of animals analyzed in each group. In accordance with the 3R (replacement, reduction, and refinement) principles [27], we used three Ch-RMs per group in an 8-week test for AKQ treatment. To minimize the experimental variables, we carefully controlled some animal characteristics, such as age and weight, in our study.

## Conclusion

AKQ reduced plasma viral loads in the SHIV89.6-infected Chinese rhesus macaque model.

## Additional files

10.1186/s13020-016-0105-x Ethical approval for research in SHIV89.6-infected monkeys.
10.1186/s13020-016-0105-x The ARRIVE guidelines checklist.

## Abbreviations

ART: antiretroviral therapy
HAART: highly active antiretroviral therapy
AKQ: *Aikeqing*
Ch-RMs: Chinese-origin rhesus macaques
HIV: human immunodeficiency virus
SIV: simian immunodeficiency virus
SHIV: simian-human immunodeficiency virus
