# Supplementary material for: Aikeqing decreases viral loads in SHIV89.6-infected Chinese rhesus macaques
Source: Chin Med. 2016 Jul 1;11:31. doi: 10.1186/s13020-016-0105-x (PMC4930622; doi:10.1186/s13020-016-0105-x)
Supplement: Supplementary file 1 — 10.1186/s13020-016-0105-x Ethical approval for research in SHIV89.6-infected monkeys. [file 13020_2016_105_MOESM1_ESM.pdf]

中国科学院昆明动物研究所

生物医学伦理委员会审批表

编号（伦理委员会根据需要编写）：SWYX—2011020

**项目名称**	**抗 HIV 中药复方优化及中医动物模型研究**
**起止时间**	2009 年 09 月至 2011 年 12 月
**实验类型及说明**	中药体内抗 SHIV 研究
**申请人及联系方式**	张高红 中国科学院昆明动物研究所 分子免疫药理学组 0871-65126855
**参与本实验的其它研究人员**	张高红，韩建保，朱林，张喜鹤，罗荣华，陈鑫，郑永唐

附件材料

附件材料：研究项目实施方案

申请人承诺：

本表及附件所包含的内容属实，如获批准，我将严格按照批准的方案进行研究，并遵守伦理委员会的相关规定。

申请人签字：[illegible]

学科组负责人签章：[illegible]

日期：2011 年 2 月 11 日

伦理委员会审批意见：

经审查，该项目符合动物医学伦理规范的相关规定，同意开展执行。

主任委员（签章）：[illegible]

中国科学院昆明动物研究所

生物医学伦理委员会（盖章）

日期：2011 年 2 月 11 日

May 13, 2014

To whom it may concern:

This is to confirm that the research protocol of the study entitled "**Chinese medicine Aikeqing decreases viral load in SHIV89.6 infected Chinese rhesus macaques**" (corresponding authour: Prof. Yong-Tang Zheng), which has been reviewed and approved by the internal review board of Kunming Institute of Zoology, Chinese Academy of Sciences (approval number: SWYX-2011020 , approval date: Feb. 11, 2011).

Sincerely

Prof. Ren Lai
Director
Internal Review Board

Kunming Institute of Zoology
Chinese Academy of Sciences

中国科学院昆明动物研究所生物医学伦理委员会审批表

附件材料：研究项目实施方案

申请日期：2011 年 2 月 11 日

项目名称： 抗 HIV 中药复方优化及中医动物模型研究

资助者类型： ☒国家 □省部 □其他：

一、项目研究目的及实验使用动物数量的必要性

由于药品资源等的限制，中医药治疗艾滋病已经成为我国部分偏远地区的重要替代疗法。临床研究发现中医药能够提高患者的生存质量，有效改善临床的部分症状，提高患者免疫力。而中医的传统诊疗手段在一定程度上限制了中药现代化，采用标准的评价指标对中药进行治疗评价能够为临床提供更有说服力的临床数据。由于恒河猴的生理、代谢、免疫等多个方面与人类最为类似，大量数据表明迄今中国恒河猴是 AIDS 研究的比较理想的模型动物，SHIV 感染的恒河猴模型目前被广泛的用于 HIV 药物和疫苗研究。利用恒河猴模型动物进行艾可清体内抗病毒活性检测，能够更好的实现实验的可控性，有效避免了临床实验中由于病毒感染时间差异等不确定因素造成的影响，更好的反应中药的疗效。实验设计遵循 3R 原则，采用两个不同剂量组和对照组三组动物，每组 3 只，共 9 只动物进行实验。本实验旨在建立和优化中药治疗 SHIV/猕猴模型研究平台，探讨复方中药艾可清在抑制病毒复制和促进 CD4+T 细胞免疫重建中的作用效果。通过对艾可清在 SHIV 感染模型中的治疗效果评价，提高艾滋病中药的合理使用、为中医药防治艾滋病奠定实验基础提供临床数据。

二、实验所需动物情况

编号	动物编号	动物品系	特殊要求	动物来源	动物饲养场所
1	#03057	中国猕猴	4-8 岁，雄性，5-10	昆明动物	攻毒前饲

2	#04331	(Chinese rhesus macaque, Macaca mulatta)	Kg，体检无浅表淋巴结肿大，经血清学检测SIV、SRV和STLV-1抗体阴性，结核菌素阴性，痢疾菌阴性。	所灵长类实验动物中心	养于实验动物中心，攻毒后饲养于ABSL-3 (Room 617) 实验室
3	#06403				
4	#01089				
5	#05077				
6	#06311				
7	#02067				
8	#06003				
9	#06025				

三、　请说明实验中所进行动物实验内容、方法、剂量与步骤。

1. 实验内容与方法：

（1） 9只猕猴随机分3组，每组3只，感染11周进入慢性感染期后，采用鼻饲管插鼻术胃部给药，持续给药8周。停药后持续观察30周；

（2）猕猴感染：采用1000 TCID50 SHIV89.6病毒株（CEMx174细胞培养）右后肢静脉攻毒途径感染中国猕猴，持续监测感染11周，进行给药实验；

（3）检测指标：每周记录体重，每天观察精神、食欲、粪便情况，试验期间死亡动物做尸检。每周采血一次，血浆进行实时定量RT-PCR病毒载量检测，全血进行流式细胞仪T淋巴细胞绝对数量分析；

（4）病毒载量检测：实验通过实时定量PCR的技术检测血浆中病毒载量，表示为每毫升血浆中含多少拷贝数的HIV-RNA，通过此项指标可以了解机体内血浆中游离病毒的水平。病毒载量是病毒复制和机体免疫清除机制共同作用的结果,反映血浆中的病毒浓度,在判断疾病进展中是一项很重要的指标,也是抗病毒药物选择和疗效的关键指标之一。分别在给药前，给药后2周、4

周、6 周、8 周，停药后 2 周，6 周，10 周，20 周，30 周进行持续观察，检测血浆中病毒载量变化；

（5）CD4 细胞数量变化：CD4+ 细胞是艾滋病病毒侵入人体后主要攻击的靶细胞,CD4+ T 细胞大量丢失，导致机体免疫功能下降，感染者免疫功能遭到严重破坏,最终机体发生免疫缺陷继而发生各种机会性感染或肿瘤。分别在给药前，给药后 2 周、4 周、6 周、8 周，停药后 2 周，6 周，10 周，20 周，30 周进行持续观察，检测模型中淋巴细胞亚型 CD3+CD4+T 淋巴细胞,及 CD3+CD8+T 淋巴细胞绝对数量变化。

2．实验流程与步骤

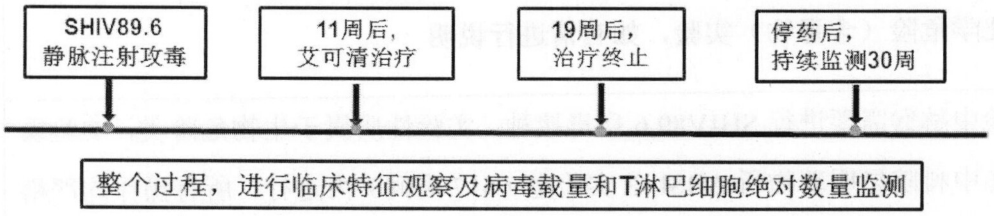

四．请说明实验中涉及到动物福利与伦理的操作，例如动物固定、投药、注射、麻醉、手术及术后照顾等

（1）人员资格审查：实验中所有操作人员均经过生物安全培训并取得了 ABSL-3 操作许可证，并具备丰富的 ABSL-3 操作经验；动物从业人员均获得云南省实验动物操作资格证及 ABSL-3 操作许可证，具有丰富的实验动物饲养操作经验和一定的生物安全操作经验。

（2）动物饲养：动物饲养在 ABSL-3 实验室，由获得实验动物操作资格证的专业人员饲养和进行日常观察；

（3）采血：所有血液采集工作均在动物完全麻醉状态下进行，采用 10 mg/kg 氯胺酮肌肉注射进行麻醉处理；

（4）安乐死处理：如果实验中动物出现持续腹泻，且体重下降 50%，拟对动

物执行安乐死处理。拟采用国际通用（American Veterinary Medical Association）的麻醉剂过量注射法处理：20mg/kg 氯胺酮肌肉注射及 80 mg/kg 戊巴比妥钠静脉注射。

五、说明实验结束后动物的处置方式（如安乐死及尸体处理方法）

由于实验结束后，动物还要进行持续的观察和监测，因此不涉及安乐死和尸体处理问题。

六、有无进行危险性实验，如生物危险（含感染性物质、致癌药物）、放射性及化学危险（含毒物）实验，如有请进行说明

实验中猕猴需要进行 SHIV89.6 病毒接种，实验性质属于生物危险类，实验操作在中科院昆明动物所 ABSL-3 实验室（617 房间）内进行，所有操作均严格按照研究所 ABSL-3 标准操作规范进行，实验人员均获得了 ABSL-3 操作许可及通过资格审查；实验废弃物采用生物安全实验室的固定设备进行高压灭菌处理后，严格按照生物安全实验室废弃物处理方法进行处理。

七、 申请人承诺

申请人保证以上所填资料完全属实。保证项目在执行中完全遵守国家《实验动物管理条例》和《医学实验动物管理实施细则》以及中国科学院昆明动物研究所实验动物使用和管理委员会章程。

申请人（PI）签名：[illegible]

日期：2011 年 2 月 11 日
